# Osteoarthritis as an evolutionary legacy: Biological ageing and chondrocyte hypertrophy

**DOI:** 10.1016/j.ocarto.2025.100624

**Published:** 2025-05-13

**Authors:** Peter M. van der Kraan

**Affiliations:** Radboudumc, Rheumatology, Experimental Rheumatology, PO Box 9101, 6500 HB Nijmegen, the Netherlands

**Keywords:** Osteoarthritis, Endochondral ossification, Chondrocyte hypertrophy, Ageing, Evolution

## Abstract

**Objective:**

Osteoarthritis (OA) is a progressive joint disease habitually linked to ageing, characterized by the gradual breakdown of cartilage leading to pain and reduced mobility. Historically viewed as mainly a “wear and tear” condition, new insights suggest that OA may be part of an evolutionary, age-related biological process rather than mainly driven by mechanical damage.

**Design:**

This conceptual paper discusses the model of antagonistic pleiotropy that proposes that certain genes beneficial early in life may contribute to diseases in the context of OA.

**Results:**

Findings indicate that OA is connected to biological and not to chronological age supporting the idea that OA is not merely a wear and tear process. Chondrocyte hypertrophy, essential in endochondral bone formation at a (pre)reproductive age, is stimulated by a displaced and wrongly timed endochondral ossification quasi-program in age-related OA. Age-related chondrocyte hypertrophic differentiation in articular cartilage is likely driven by loss of loading-induced TGF-β signaling.

**Conclusion:**

Comprehending OA within this evolutionary and biological frame provides a solid alternative to the theory of “wear and tear”, offering insights into further understanding, prevention and disease management.

## 1 Introduction

This is a narrative review/conceptual article inspired by recent publications by Gems et al. and our own previous work to better understand the pathobiology of age-related osteoarthritis (OA) [[1], [2], [3], [4]], in an effort to move toward a better understanding of the how and why of OA.

Osteoarthritis can, in a simplified version, viewed in two ways. First, an age-related process of cartilage destruction and joint remodeling and second, and in a clinical and patient-oriented view, as a process leading to joint dysfunction, pain and other symptoms. In OA, joint destruction and pain are interconnected but not one to one coupled [5]. In this article, OA will be discussed in the first part as a process of joint destruction whilst in the last part the clinical picture of the disease will be integrated.

### 1.1 Osteoarthritis, ageing and evolution

Osteoarthritis is a heterogeneous and invalidating chronic disease of the joint. It is strongly associated with ageing and characterized by gradual joint deterioration leading to moderate to severe pain affecting almost all daily activities of patients. It is an individual, societal and economic burden that will strongly increase in the near future. In 2020 the number of people worldwide with OA was estimated by the World Health Organization to be 595 million and expected to grow to 1100 million in 2050 [6].

Old age and OA are linked. Ageing is the functional deterioration of an organism ultimately ending in death. Theories of ageing have two extremes, at one end accumulation of damage that leads to dysfunction and death, and at the other end ageing as a programmed and defined biological process, with many hypotheses in between [7].

**Fig. 1:**
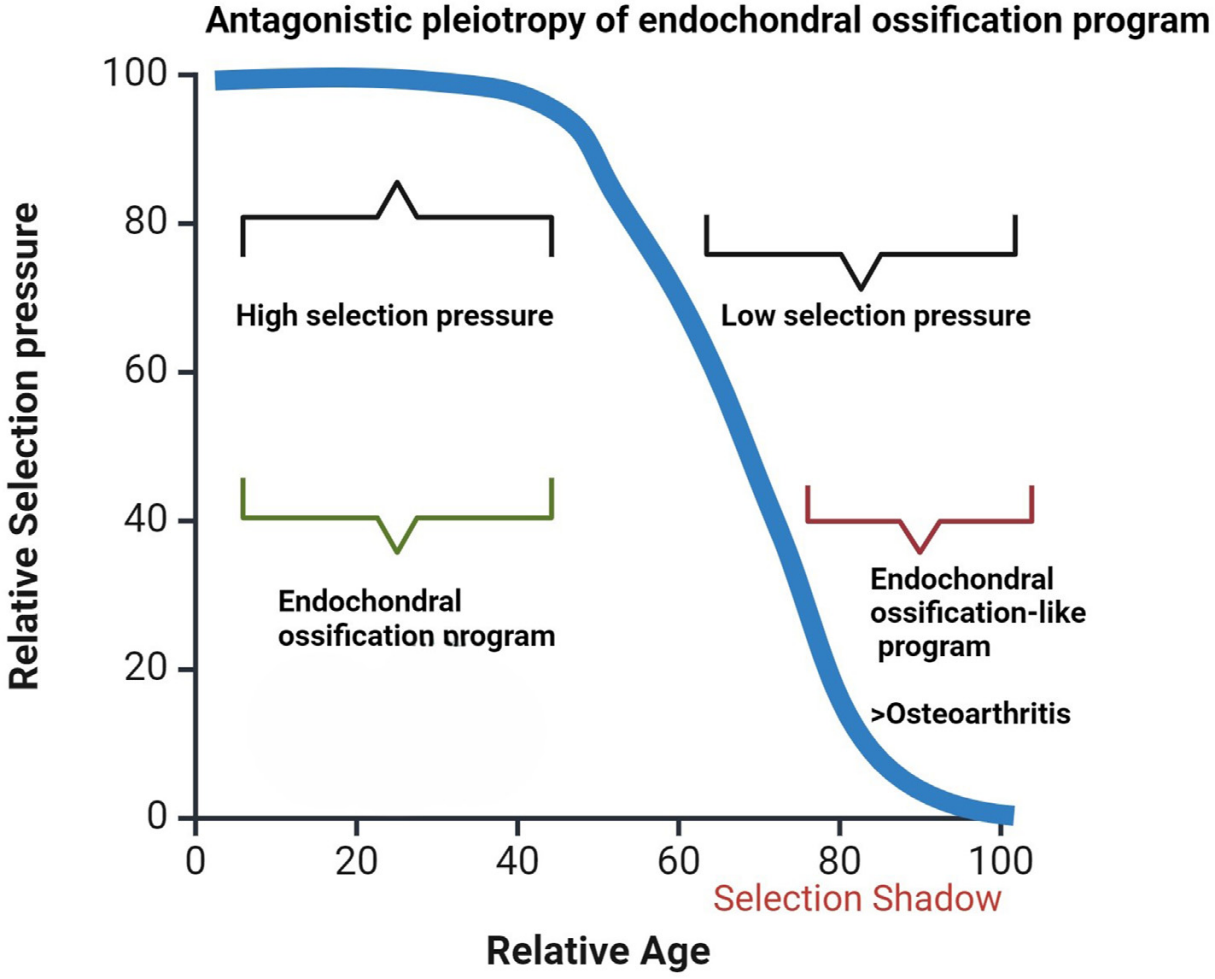
Antagonist pleiotropy of the endochondral ossification (quasi)-program. Antagonistic pleiotropy is the concept that genes and processes that enhance fitness early but reduce it later life can still be favoured by natural selection. Even genes that are deleterious in late life can be positively selected for and maintained in the population when having an advantage at the reproductive age.

An alternative explanation for the ageing processes is antagonistic pleiotropy [8]. This postulates that genes and processes that enhance fitness (survival and reproduction) early in life but reduce it later life can still be preferred by natural selection (Fig. 1), because selection is strong early and weak late in life (selection shadow). As a consequence, even processes and genes that are deleterious in late life can be positively selected for and maintained in the population when having an advantage at a younger age. As a result, these genes can be the cause of afflictions that occur later in life [1,8]. Examples are cancer, coronary heart disease and Alzheimer's disease [9]. Osteoarthritis is a general phenomenon in “higher” vertebrates and should be considered in the context of evolution. To quote Theodosius Dobzhansky “*Nothing in Biology Makes Sense except in the Light of Evolution*”(10). Therefore, OA should be considered as a process related to biological age and be executed by an evolutionary maintained “biological program”, that is beneficial early but harmful late in life.

The observation that the main risk factor for OA is age, in combination with an engineering view on joints and articular cartilage, has led to view OA as driven by a mechanical “wear and tear” process. The more it is used the more it will wear down. Deliberating this, it has be considered that OA is not a human specific affliction. Many non-human animals, in all probability all well studied mammals, reptiles and birds, are reported to develop OA. OA is found in terrestrial mammalian species, such as elephants, dogs, mice, monkeys, primates but also in water-bound species like whales. Birds develop OA irrespective of their size and posture and also in reptiles, such as turtles, OA is reported [[11], [12], [13]]. Animals, including humans, as a species develop OA in general at a relatively similar biological age, some examples; Human sapiens, OA 50–60 years, lifespan expectancy: 80–85 years, mouse (*Mus musculus*), OA 12–14 months, lifespan: 18–24 months, cat (*Felis catus*) 11–12 year, life expectancy 15–17 years, dogs (*Canis lupus familiaris*) OA 9–10 years, life expectancy 13–15 years.

Concluding, OA appears to hold pace with life expectancy of a species and not with chronological age. It is hence highly unlikely that OA is a simple process of mechanical wear and tear, but that it is linked to biological ageing [3]. It should be looked upon as an age-related affliction that leads to dysfunction, pain and other symptoms, comparable with other age-related diseases such as coronary heart disease and dementia, and should also be approached as such.

### 1.2 Accelerated biological ageing and osteoarthritis

Chronological age is the number of months or years an individual has lived while biological age is a physiological parameter that can be altered by a range of factors. Individuals of the same chronological age can have markedly different biological age, with some ageing at a much faster pace than others. Lifestyle, including diet, physical activity, smoking and alcohol consumption, are associated with estimates of biological age [14,15]. Beneficial lifestyle factors that slow down biological ageing are also associated with lower odds of OA [16]. In a study with over 30.000 participants findings indicated that an increase in parameters of biological ageing were associated with a higher risk of OA compared to the risk of participants with low levels of these parameters [17]. This shows, also in the light of what has been discussed above, that OA is closely related to biological ageing.

Chronic metabolic disease is lifestyle-related and has a high incidence in the Western World [18]. Chronic metabolic disease is known to accelerate biological ageing and increases the risk of chronic diseases [19]. Holmannova et al. showed that people with metabolic syndrome have higher levels of markers of biological aging than controls [20]. These findings show that accelerated biological ageing, linked to the metabolic syndrome, is associated with an increased risk of chronic comorbidities like cardiovascular and neurodegenerative diseases and OA. In this line of reasoning, it is not surprising that people with metabolic syndrome have a higher prevalence of OA [21,22].

Mice (*Mus musculus*) are extensively used as animal models of human disease and also these suffer from age-associated OA. Mouse models that speed up ageing, such as Senescence-accelerated mouse-prone 8, UM-HET3, G608G, zinc metallopeptidase, STE24 (ZMPSTE24) and Klotho mice, show earlier OA-like changes in their joints than their normal counterparts [[23], [24], [25], [26], [27]]. Interestingly, Snell dwarf mice show, in addition to increased longevity, delay of OA development [28]. However, not in all mouse models of speeded ageing premature OA has been reported. The mouse model of the human progeroid DNA repair trichothiodystrophy syndrome do not show OA at a relatively early age [29]. In general, in mouse models of accelerated ageing early OA is common, but not always. Physiological differences between the ageing mechanisms in these models, not all elucidated yet, most likely underly these differences. Overall, factors that accelerate biological ageing result in premature development of OA, further indicating that not wear and tear is the underlying cause of OA but a biological age dependent “biological program”.

As we postulated earlier, osteoarthritis is a universal biological phenomenon in mammals, birds and reptiles with a biological foundation that should be relevant in the light of evolution [3,10]. We hypothesized that the basic biological process is increased hypertrophy of articular chondrocytes activating endochondral ossification-like processes at a time and location that ordinarily should be deprived of this [3,4]. Furthermore, we have elucidated that loading-induced active Transforming Growth Factor- β (TGF-β) signaling normally acts as an important block on chondrocyte hypertrophy in articular cartilage [30,31].

Endochondral ossification that has strong advantages early in human and animal life, is a non-adaptive developmental (semi) program later in life, an endochondral ossification quasi-program. Hypertrophic articular chondrocytes start non-functional joint remodeling at the wrong time at the wrong place. This concept makes the story of the “how and why” of OA more complete as an evolutionary founded process. However, it should be recognized that underlying this process multiple factors will act activating, accelerating, and modulating. Further, when started and progressing a whole cascade of events will contribute to structural joint damage, remodeling and symptoms.

### 1.3 Endochondral ossification, chondrocyte hypertrophy and osteoarthritis

Cartilage is present in the body in a number of varieties, hyaline, fibro and elastic. Hyaline cartilage can be found in the growth plate and at the end of long bones, articular cartilage. Growth plate cartilage being temporary and articular cartilage permanent. Growth plate cartilage is replaced by bone (endochondral ossification), with a key role for hypertrophic chondrocytes, while articular cartilage is inhibited to take this path under healthy conditions. The default route of chondrocyte differentiation in this lineage is considered to be endochondral ossification [32,33].

Hypertrophic chondrocytes are found in many skeletal structures, the growth plates, the calcified layer of articular cartilage and during fracture healing. Hypertrophic chondrocytes are characterized morphologically as enlarged chondrocytes. They specifically express *Col10a1*, encoding type X collagen, a key marker of hypertrophic chondrocytes.

**Fig. 2:**
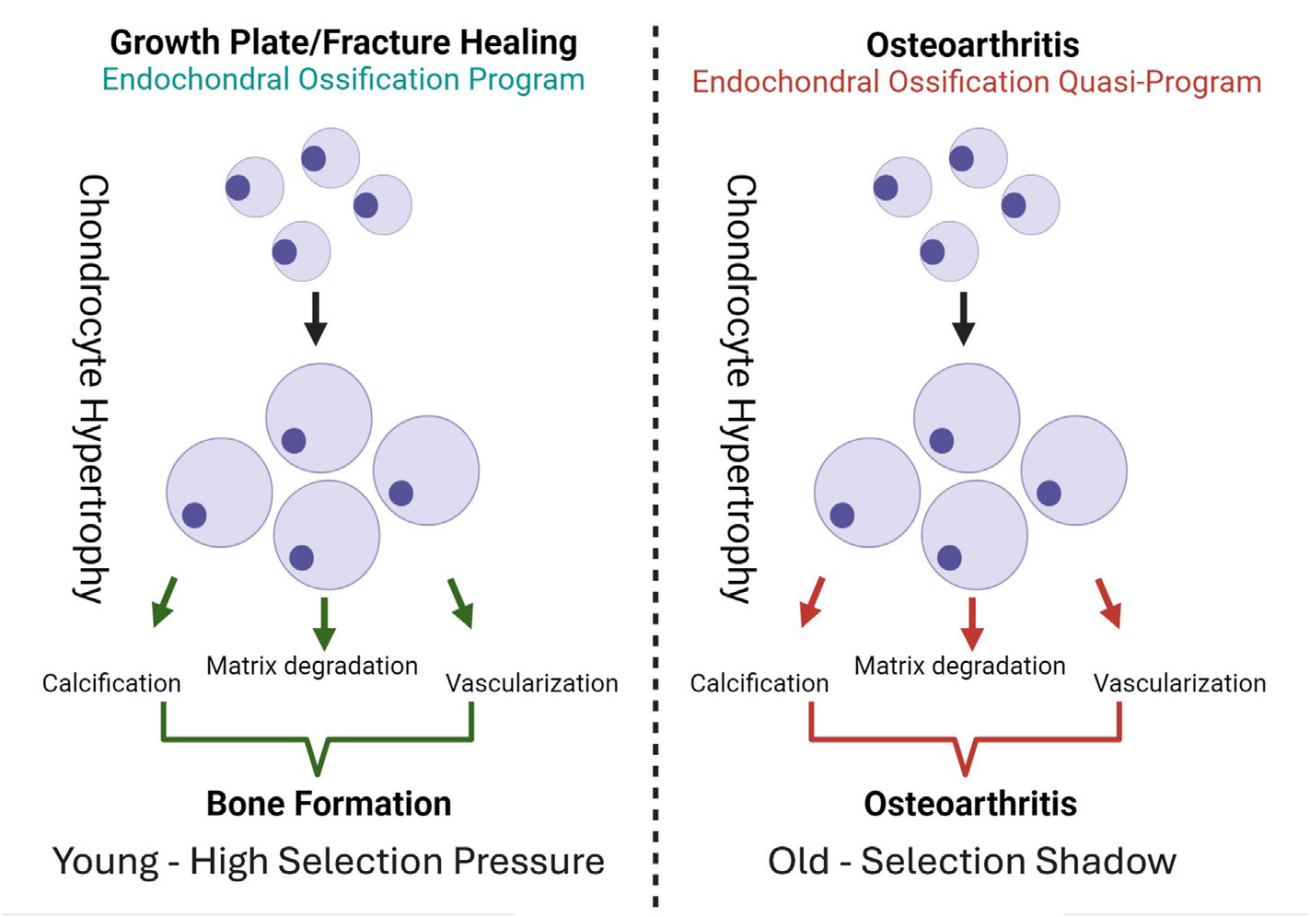
The endochondral ossification program and the endochondral ossification quasi-program. Both the endochondral ossification program and the endochondral ossification quasi-program lead to cartilage matrix degradation, vascularization and calcification that can be observed in the growth plate, fracture healing and OA.

Hypertrophic chondrocytes express Vascular Endothelial Growth Factor which induces vascularization of the healing bone [34]. The degradation and resorption of cartilaginous tissue by hypertrophic chondrocytes is mainly carried out by matrix metalloproteinases, in particular MMP13 and MMP9 [35,36]. Furthermore, hypertrophic chondrocytes can differentiate into osteoprogenitors, contributing directly to bone formation. The cartilage-to-bone transition in the growth plate and during fracture healing consists of multiple events including the degradation of the cartilage matrix, vascular invasion, and calcification, phenomena that strongly resemble the processes observed in articular cartilage in OA (Fig. 2) [37].

A fundamental process that occurred relatively early during evolution might be at the base of the process early in life that is of advantage in the context of antagonistic pleiotropy to increase fitness [2]. The evolution of endochondral bone formation is thought to have occurred in the evolutionary lineage of the osteichthyans, a lineage that has led to modern bony fish, amphibians, reptiles, bird and mammals [38]. The process of endochondral ossification has occurred simultaneously with evolving of the first synovial joint in osteichthyans, the jaw [39,40]. This evolutionary lineage originating from and including the osteichthyans has been shown to be very successful, strongly indicating that having these traits contributed to evolutionary success. Osteoarthritis is ubiquitous and development has shown to occur already in the jaw joint of ageing zebrafish [40]. Therefore, although modern fish and humans diverged hundreds of millions of years ago they share similar OA susceptibility of synovial joints revealing that both synovial joints and osteoarthritis are as old as endochondral bone itself [40]. With this in mind, one might conclude that the process of endochondral bone formation early in life outweighs the deleterious process of a similar quasi-process late in life that leads to OA in the selection shadow.

### 1.4 Loading, unloading and chondrocyte hypertrophy

Growth plate cartilage is temporary, as is the callus in healing fractured bones, while articular cartilage is in principle permanent. To be maintained, articular cartilage needs to be loaded and unloading leads to loss of cartilage. Patients with spinal cord injuries show progressive loss of knee cartilage at a faster rate than OA patients [41,42]. Moreover, in a rat knee immobilization model elevated chondrocyte hypertrophy and increased expression of metalloproteinase-13 was seen in the articular cartilage [43,44].

TGF-β is known to inhibit hypertrophic differentiation of chondrocytes and its production and activation is in articular cartilage increased by mechanical loading [[45], [46], [47]]. We have demonstrated that loading rapidly induces TGF-β signaling in healthy articular cartilage, as measured by expression of phosphoSMAD2/3, most likely due to mechanical activation of latent TGF-β bound to cartilage matrix. Unloading results in loss of signal within hours [30]. Prolonged unloading (2 weeks) led to strongly increased *Col10a1* expression, that could be prevented by *in vitro* loading of the cartilage every three days. The conclusion that loading prevents endochondral ossification in cartilage is confirmed by the observation that unloaded cartilage of rat knee joints immobilized in flexion turn rapidly within weeks into bone [48,49].

The loading activated SMAD2/3 signaling induced a positive feedback loop, by stimulating expression of *Tgfb1* and the type I TGF-β receptor *tgfbr1* (*Activin* Receptor-like Kinase-5 (ALK5), SMAD2/3 route) and decrease of the alternative TGF-β receptor *acvrl1* (ALK1, SMAD1/5/8 route). The Bone Morphogenetic Protein (BMP)-related SMAD1/5/8 route is known to stimulate hypertrophy and endochondral ossification. All aspects of loading induced TGF-β signaling could be blocked by an ALK5 (TGF-β type 1 receptor) inhibitor. Importantly, old cartilage shows a strongly reduced capacity for this loading mediated TGF-β-SMAD2/3 signaling compared to young, which will make it more susceptible to OA development fitting with the age-related nature of OA [50].

The technique of joint distraction is an interesting technique that can delay the period to knee joint replacement in relatively young people [51]. At first glance, this might be contradictory with the proposed concept in this article. However, the concept proposed here is relevant to the initiation of the OA process while joint distraction is applied in knee joints with established OA. Joint distraction most likely initiates a stem cell dependent repair process [51]. Furthermore, joint distraction is not immobilization of the joint, a certain amount of movement is still possible and necessary. It has been shown that levels of TGFβ1 are significantly increased in the synovial fluid in most patients undergoing this procedure [52] Moreover, enhanced expression of TGFβ receptor type 1 (TGFBR1) and TGFBR2 is observed in synovial fluid-mesenchymal stem cells during early distraction treatment. This is thought to contribute to the reparative response seen during the distraction treatment [53]. TGFβ is a well-known stimulator of chondrogenic differentiation in mesenchymal stem cells [54,55]. In conclusion, in both processes TGFβ is appears to be involved, in normal cartilage to prevent the initiation of the OA process by inhibiting hypertrophic chondrocyte differentiation and during joint distraction by stimulating a mesenchymal stem cell-based repair process.

Loading is necessary to keep articular cartilage healthy and unloading results in excessive chondrocyte hypertrophy due to the absence of the block on the progression of chondrocyte hypertrophic differentiation. Interestingly, it is well-known that excessive motion of fractured bone ends results in nonunion of these fractures, failing endochondral ossification. Although little research has been performed on the biological processes important in nonunion, strongly elevated expression of TGF-β in nonunion fracture compared to stable fractures has been reported, possibly contributing to insufficient chondrocyte hypertrophy [56].

It is a simplified view that endochondral ossification and chondrocyte hypertrophy is only regulated by TGF-β. Many factors are involved that can be either inhibitory or stimulatory. Well known examples are Wingless-related integration site and BMPs, factors not only involved in regulation of chondrocyte hypertrophy but also shown to play a role in OA [[57], [58], [59], [60], [61]]. Other factors that are implicated are discussed by Mackie et al. and Hallett et al. [62,63].

For example, Indian hedgehog (Ihh) signaling is essential in chondrocyte hypertrophic differentiation [64]. It is reported that blocking ihh signaling prevents joint destruction in OA mice, which was associated with a decrease in OA markers [65,66]. However, other authors that interfered with ihh signaling did not show a protective effect on OA in a murine post-traumatic OA model [67]. It has to be kept in mind that post-traumatic OA models in mice are not age-related OA models, but surgically induced, and inflammation is an integral part of the induction of these models due to the insulted tissue damage. For instance in the latter study, although collagen type X mRNA expression was reduced, expression of MMP13 mRNA and protein was not significantly altered. In the in this paper proposed concept chondrocyte hypertrophy leads to damage via, amongst others, synthesis of MMP13 while in post-traumatic OA models inflammation can be a driving force of MMP13 production.

Reactivation of an endochondral ossification process is not the whole story underlying OA, trauma to cartilage and bone leads to damage and inflammation, but likely also to the activation of repair processes including endochondral ossification. Furthermore, it can be imagined that various aberrant events and processes in the joint, and particular in the cartilage-bone unit might initiate and or accelerate an endochondral ossification quasi-program and chondrocyte hypertrophy.

### 1.5 Disease expression and clinical symptoms

There are major individual differences in development and progression of OA. Not everyone will be as sensitive to aberrant activation of the endochondral ossification quasi-program, and this program might be activated at different ages or not all at all during a lifetime.

Moreover, activation of this quasi-program will not in all cases lead to clinical OA. A certain level of cartilage destruction can be present without symptoms. Also, it is known that the correlation between destruction and symptoms can be poor at an individual level. For instance, a considerable proportion of patients with radiographic knee OA do not show regular symptoms, or have no symptoms at all [68]. Genetics, lifestyle, life events and chance will all be of influence on both initiation and progression of the program and symptoms.

Although it is likely that reactivation of an endochondral ossification-like program lies beneath the age-related structural changes in OA articular cartilage and succeeding changes in the joint, other factors will be driving forces of OA initiation, progression and symptoms. All that stimulates endochondral ossification-like processes in a joint could accelerate the initiation of OA. A major factor will be the genetic background of an individual and an association between proteins and pathways involved in endochondral ossification and OA has been proven. In a genetic screen of over 800.000 people including 177.000 with OA, approximately 70 high confidence effector genes ordinarily involved in bone formation have been identified as OA risk genes, amongst those Growth Differentiation Factor-5 (*GDF5)* and *SMAD3* with the highest levels of confidence [69]. Furthermore, joint trauma will frequently lead to damage of cartilage and bone leading to a reparative response resembling endochondral ossification. Intra-articular fractures contribute significantly to OA of the knee, reported is that 23 ​%–44 ​% of those fractures leads to OA [70,71]. Even joint trauma in which cartilage and bone is not primarily involved, e.g. Anterior cruciate ligament (ACL) trauma, will lead to metabolic changes in cartilage and bone. Interestingly, more and more it is postulated that not overloading but underloading is involved in cartilage damage after ACL rupture, even when surgically repaired. This might be related to decreased TGF-β signaling and loss of the block on activation of an endochondral ossification quasi-program [31,72,73]. Tissue trauma in the joint, possibly also repetitive microtrauma, will lead to an inflammatory reaction and released inflammatory factors can contribute to OA via stimulation of the mentioned program [74,75]. How and when the endochondral ossification quasi-program is modulated will not only affect the initiation of this process but also the progression, and will determine if, when and how this will run in an individual.

In general, initial consequence of the activation of an endochondral ossification quasi-program will not lead to symptoms. In people with radiographic knee OA the proportion with pain ranged from 15 ​% to 81 ​% [5]. Pain in OA is associated with a structural changes including the presence of bone marrow lesions, synovitis and intra-articular mineralization but poorly with cartilage destruction [76,77]. Symptoms are not caused by the early stage of the underlying basic process but related to later joint changes, such as bone remodeling and synovitis, in combination with systemic regulatory processes of pain, personal predisposition and environmental and psychosocial factors [78].

## 2 Discussion and conclusion

The considerations above are focused on the large group of OA patients in which the main risk factor is age. Of course, in specific cases other factors will be the main driving force of the disease. Regarding this, mutations that interfere with the primary function of articular cartilage will lead to early OA [79,80]. Still, even in these specific cases a role for activation of the endochondral ossification quasi-program might be involved, for instance ACL rupture-dependent post-traumatic OA or in patients with *SMAD3* mutations [81].

Is the concept of an evolutionary-founded general underlying processes In OA, not being a process of wear and tear, relevant for OA patients? It can be expected that better understanding of OA provides tools to improve explanation of the disease. The why and the consequences of sufficient physical activity to prevent and treat OA can be better communicated to the general population and OA patients. Unfortunately, it has to be acknowledged that the idea that physical activity wears down the cartilage of your joints is still a common one, and patients with OA often limit their physical activity due to kinesiophobia [82].

A better understanding of OA will also focus the development of pharmacological therapy to treat OA. Treatment of symptoms, like pain, will remain essential since the features of OA still lead to late detection of the OA. However, inhibition of progression, especially in the early stage, might benefit by the development of drugs that interfere with advancement of a endochondral ossification quasi-program.

Recently, many efforts to stratify OA patients in phenotypes and endotypes are performed and still ongoing. Although a common process might be at the root of OA, how this is initiated, progressing and how symptoms are displayed will differ between patient groups. It is especially important to elucidate what is the best treatment for a specific patient group, the best treatment for the right patient. The concept of theratypes that has been recently proposed to stratify OA and to focus treatment development might be supportive in this respect [83].

In a large part of the general population development of OA is associated with an age-related reactivation of a displaced endochondral ossification quasi-program in articular cartilage. Reactivation of this program can be seen in the framework of pleiotropic antagonism, a principle that explains the occurrence of harmful processes in the period of life of an individual in the “selection shadow”. Occurrence and pace of this process, and expression of OA symptoms, is modulated by inherent, lifestyle and life events. Understanding OA within an evolutionary and biological framework provides a robust alternative to the theory of “wear and tear” offering insights into its management and prevention.

## Author contributions

PvdK is responsible for all aspects of this publication.

## Declaration of competing interest

Pvdk has no conflicts of interest with regards to this publication.
